# Energy Metabolism and Stemness and the Role of Lauric Acid in Reversing 5-Fluorouracil Resistance in Colorectal Cancer Cells

**DOI:** 10.3390/ijms26020664

**Published:** 2025-01-14

**Authors:** Rina Fujiwara-Tani, Yi Luo, Ruiko Ogata, Kiyomu Fujii, Takamitsu Sasaki, Rika Sasaki, Yukiko Nishiguchi, Shiori Mori, Hitoshi Ohmori, Hiroki Kuniyasu

**Affiliations:** 1 Department of Molecular Pathology, Nara Medical University School of Medicine, Kashihara 634-8521, Japan; lynantong@hotmail.com (Y.L.); pkuma.og824@gmail.com (R.O.); toto1999-dreamtheater2006-sms@nifty.com (K.F.); takamitu@fc4.so-net.ne.jp (T.S.); rika0st1113v726296v@icloud.com (R.S.); yukko10219102@yahoo.co.jp (Y.N.); m.0310.s.h5@gmail.com (S.M.); brahmus73@hotmail.com (H.O.); 2 Department of Cancer Biology, Institute of Biomedical Science, Kansai Medical University, Osaka 573-1010, Japan

**Keywords:** 5-fluorouracil, drug resistance, colorectal cancer, oxidative stress, energy metabolism, cancer stemness

## Abstract

While 5-fluorouracil (5FU) plays a central role in chemotherapy for colorectal cancer (CRC), resistance to 5FU remains a major challenge in CRC treatment, and its underlying mechanisms remain unclear. In this study, we investigated the relationship between 5FU resistance acquisition, stemness, and energy metabolism. Among the two CRC cell lines, HT29 cells exhibited glycolytic and quiescent properties, while CT26 cells relied on oxidative phosphorylation (OXPHOS) for energy. In contrast, the 5FU-resistant sublines (HT29R and CT26R), developed through continuous exposure to low concentrations of 5FU, demonstrated enhanced stemness. This was associated with glycolytic dominance, low proliferation, and reduced reactive oxygen species (ROS) production. However, treatment with the medium-chain fatty acid lauric acid shifted the cells to OXPHOS, reducing stemness, increasing ROS levels, and inducing cell death, therefore reversing 5FU resistance. These findings suggest that an enhancement in stemness and the reprogramming of energy metabolism play key roles in acquiring 5FU resistance in CRC. While lauric acid reversed 5FU resistance, further clinical studies are required.

## 1. Introduction

Various anticancer drugs have been developed to treat solid cancers and their efficacy has improved steadily over time [1,2]. This progress has been driven by advancements in drug discovery, especially through genome analysis [3]. Molecular-targeted drugs have emerged, focusing on the environmental response mechanism of cancer cells, including hypoxia [4], interactions between cancer cells and the stroma [5,6], and the delivery systems of anticancer agents [7,8]. However, developing resistance to anticancer drugs by cancer cells remains a significant barrier to long-term drug efficacy [4].

In recent years, research on anticancer drug resistance has highlighted the role of energy metabolism and stemness in cancer cells [9,10,11,12,13,14]. Multidrug resistance in cancer is often linked to cancer stem cells (CSCs), which exhibit unique resistance mechanisms, including the expression of stem cell-specific factors such as adenine triphosphate (ATP)-binding cassette transporters and aldehyde dehydrogenase, changes in the CSC microenvironment, and epithelial–mesenchymal transition [15,16,17]. Energy metabolism is intricately linked to the maintenance of CSCs. Cancer cells predominantly rely on glycolysis for energy production through the Warburg effect, facilitated by enhanced sugar uptake [18]. This increased glycolytic activity supports rapid energy production and enhances stemness through metabolites produced during glycolysis [19].

CSCs maintain their stemness through a dual strategy involving glycolysis and oxidative phosphorylation (OXPHOS) [20,21,22]. CSCs are heterologous, comprising low-proliferative CSCs (dormant CSCs or quiescent CSCs) and high-proliferative CSCs, with the former being more resistant to treatment [22,23,24].

In colorectal cancer (CRC), approximately 50% of cases are diagnosed at stage III or higher, with a 5-year survival rate of <10% for metastatic CRC [25]. Consequently, chemotherapy plays a crucial role in treating advanced CRC, with 5-fluorouracil (5FU), a single agent or in combination with various anticancer drugs, playing a central role in CRC [25,26]. However, resistance to 5FU is a major clinical challenge. The key mechanisms contributing to 5FU resistance in CRC cells include overexpression of thymidylate synthase (TS), an enzyme targeted by 5FU [27,28]; degradation of 5FU by dihydropyrimidine dehydrogenase (DPD) [29,30]; downregulation of methylenetetrahydrofolate reductase (MTHFR) [30] and thymidine phosphorylase (TYMP) [29,31], and enhanced DNA damage repair [32,33].

Recently, the relationship between energy metabolism and stemness has gained attention as a contributor to 5FU resistance. Our previous studies on gemcitabine-resistant pancreatic cancer cell lines demonstrated that metabolic reprogramming from OXPHOS to glycolysis, combined with enhanced glutaminolysis, contributes to resistance. This shift reduces mitochondrial oxidative stress to promote drug resistance [34]. Furthermore, stabilization of hypoxia-inducible factor-1α (HIF1α) enhances stemness, further contributing to gemcitabine resistance [35]. These findings suggest that energy metabolism and stemness are interdependent factors in acquiring drug resistance. However, their specific roles in 5FU resistance in CRC remain poorly understood.

Lauric acid (LAA), a medium-chain fatty acid (MCFA), has been shown to overcome gemcitabine resistance in pancreatic cancer cell lines [35]. MCFAs have characteristic metabolic properties including faster intestinal absorption and tissue transport, compared with long-chain fatty acids [36,37,38]. Moreover, they promote OXPHOS through rapid β-oxidation by carnitine shuttle-independent mitochondrial transport [38,39]. In cancer cells with mitochondrial damage, MCFAs can induce excessive mitochondrial oxidative stress and cell death [35,40,41].

In this study, we investigated the roles of energy metabolism and stemness in 5FU resistance using two types of CRC cell lines. We further investigated the potential of LAA as a modulator of 5FU resistance.

## 2. Results

### 2.1. Differences in Proliferation, Differentiation, and Energy Metabolism

**Figure 1:**
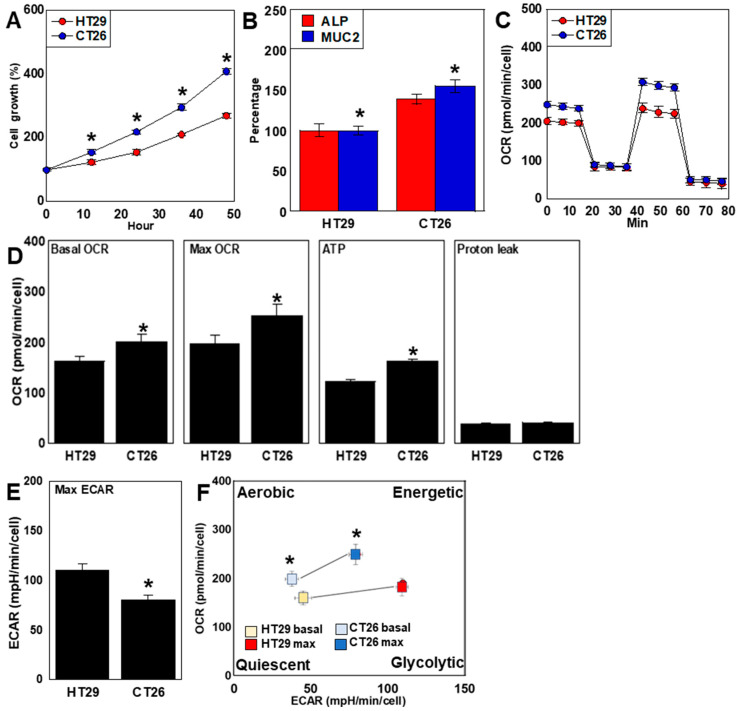
Differences in proliferation, differentiation, and energy metabolism between two CRC cell lines. HT29 and CT26 cells were cultured in a regular medium. (**A**) Cell growth. (**B**) Protein levels during colonocyte-associated differentiation. (**C**) Mitochondrial stress test results. (**D**) OXPHOS parameters. (**E**) Glycolytic stress test results, including the maximum ECAR. (**F**) Energy metabolism phenotypes. Error bars: standard deviation of three independent trials. Asterisk, *p* < 0.05. Statistical differences were calculated using ordinary ANOVA with Bonferroni correction. CRC, colorectal cancer; ALP, alkaline phosphatase; MUC2, mucin 2; OCR, oxygen consumption rate; ECAR, extracellular acidification rate; Max, maximum; OXPHOS, oxidative phosphorylation; ANOVA, analysis of variance.

Examination of two CRC cell lines, HT29 and CT26, with stemness properties different from the cell lines we previously reported [42,43] showed higher proliferation of CT26 cells compared with HT29 cells under normal culture conditions (Figure 1A). Investigation of the degree of differentiation of the two cancer cell lines into the colonic mucosal epithelium revealed higher gene expression of alkaline phosphatase (*ALP*) and mucin 2 (*MUC2*), which are colonic mucosal epithelial markers, in CT26 cells compared with HT29 cells (Figure 1B). The assessment of OXPHOS demonstrated a higher basal oxygen consumption rate (OCR), maximum OCR, and ATP production in CT26 cells compared with HT29 cells (Figure 1C,D). In contrast, glycolysis was enhanced more in HT29 cells than in CT26 cells (Figure 1E). The energy metabolism profiles of the two cancer cell lines showed that HT29 cells tended to be more quiescent than CT26 cells (Figure 1F).

### 2.2. Differences in Oxidative Stress

**Figure 2:**
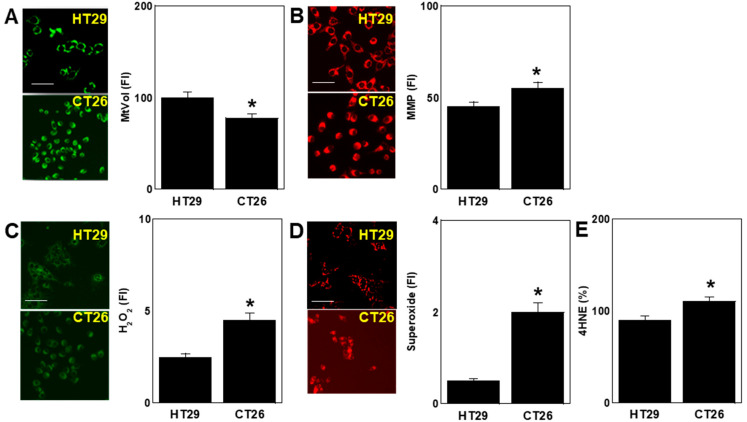
Differences in oxidative stress between two CRC cell lines. HT29 and CT26 cells were cultured in a regular medium. (**A**) Assessment of MtVol with mitogen. (**B**) MMP assessed using TMRE. (**C**) H_2_O_2_ levels assessed using DHR123. (**D**) MtSOX. (**E**) 4HNE. Scale bar: 50 μm. Right panel: semi-quantification of fluorescence images. Error bars: standard deviation of three independent trials. Asterisk, *p* < 0.05. Statistical differences were calculated using ordinary ANOVA with Bonferroni correction. CRC, colorectal cancer; MtVol, mitochondrial volume; MMP, mitochondrial membrane potential; TMRE, tetramethylrhodamine ethyl ester; DHR123, dihydrorhodamine 123; mtSOX, mitochondrial superoxide; 4HNE, 4-hydroxynonenal; ANOVA, analysis of variance.

Investigation of mitochondrial oxidative stress showed higher mitochondrial volume in HT29 cells than in CT26 cells and higher mitochondrial membrane potential (MMP) in CT26 cells than in HT29 cells (Figure 2A,B). The mitochondrial hydrogen peroxide, superoxide, and lipid peroxide (4-hydroxynonenal, 4HNE) levels were higher in CT26 cells than in HT29 cells (Figure 2C–E).

### 2.3. Differences in Stemness

**Figure 3:**
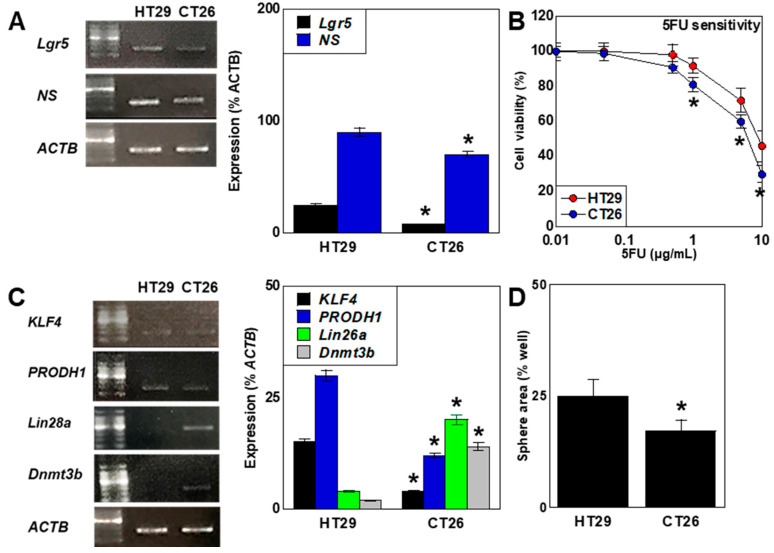
Differences in stemness between two CRC cell lines. HT29 and CT26 cells were cultured in a regular medium. (**A**) Expression of stemness marker genes *LGR5* and *NS*. Right panel: semi-quantification of RT-PCR signals. (**B**) Sensitivity to 5FU. (**C**) Expression of naïve/prime transition-associated genes *KLF4*, *PRODH*, *LIN28a*, and *DNMT3B*. Right panel: semi-quantification of RT-PCR signals. (**D**) Sphere areas. Error bars: standard deviation of three independent trials. Asterisk, *p* < 0.05. Statistical differences were calculated using ordinary ANOVA with Bonferroni correction. CRC, colorectal cancer; LGR5, leucine-rich repeat-containing G-protein coupled receptor 5; NS, nucleostemin; ACTB, β-actin; RT-PCR, reverse transcription polymerase chain reaction; 5FU, 5-fluorouracil; KLF4, Krüppel-like factor 4; PRODH, proline dehydrogenase; DNMT3B, DNA methyltransferase 3B.

Gene expression of leucine-rich repeat-containing G-protein coupled receptor 5 (*LGR5*) and nucleostemin *NS*, CRC stemness markers expressed in both cell types, was higher in HT29 cells than in CT26 cells (Figure 3A). Sensitivity to 5FU was lower in HT29 cells than in CT26 cells (Figure 3B). Pluripotent stem cells showed naïve or prime stemness, with the latter demonstrating enhanced proliferation and differentiation [44,45]. The expression of naïve markers (Krüppel-like factor 4 [*KLF4*] [46] and proline dehydrogenase [*PRODH*]) [47] was high in HT29 cells, while the expression of markers for prime stemness, *Lin28a* [48], and DNA methyltransferase-3b (*DNMT3B*) [49] was high in CT29 cells.

These results suggest that HT29 cells are less proliferative and have a glycolysis-dominated energy metabolism, thus showing naïve-like stemness. In contrast, CT26 cells are highly proliferative and have OXPHOS-dominated energy metabolism, thus showing prime-like stemness.

### 2.4. Characterization of 5FU-Resistant Cell Lines

**Figure 4:**
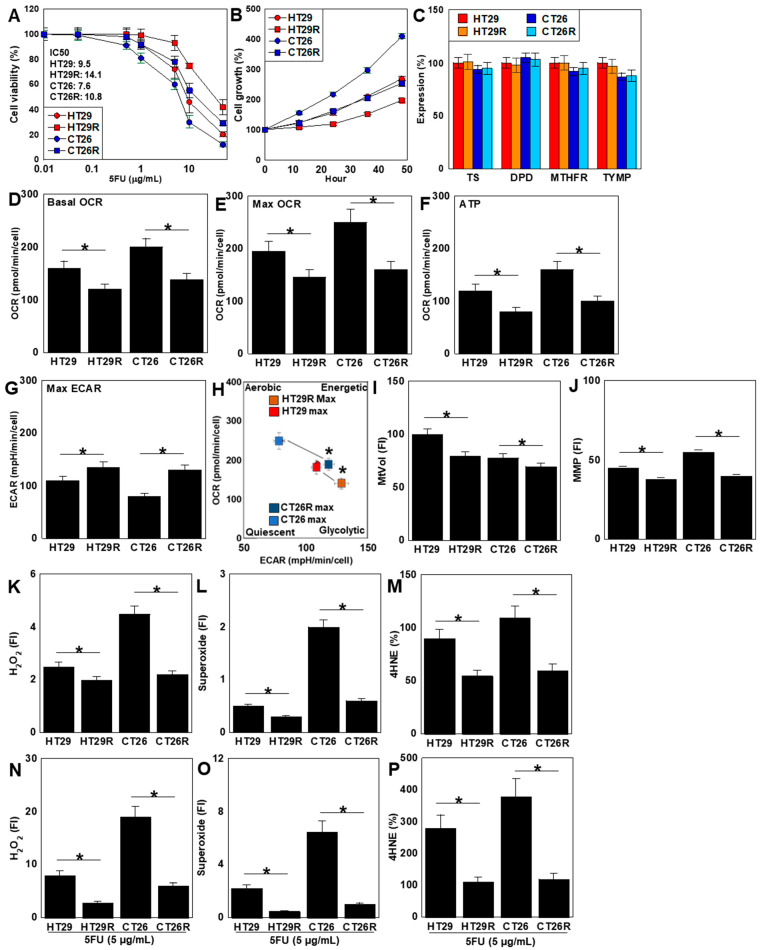
Characterization of 5FU-resistant cell lines derived from two CRC cell lines. The HT29R and CT26R cell lines resistant to 5FU were established from HT29 and CT26 cells, respectively, by continuous treatment with low-dose 5FU (IC_5_) for 50 passages. (**A**) 5FU sensitivity, indicated by IC_50_. (**B**) Cell growth in regular medium. (**C**) Expression of 5FU-resistance-related genes *TS*, *DPD*, *MTHFR*, and *TYMP*. (**D**–**F**) OXPOHS parameters: basal OCR (**D**), maximum OCR (**E**), and ATP (**F**). (**G**) Max ECAR. (**H**) Energy metabolism phenotype. (**I**) MtVol assessed using MitoGreen. (**J**) MMP assessed using TMRE. (**K**) Mitochondrial H_2_O_2_ levels were assessed using DHR123. (**L**) MtSOX. (**M**) 4HNE. (**N**–**P**) HT29R and CT26R treated with 5FU (5 μg/mL for 48 h). H_2_O_2_ (**N**), mtSOX (**O**), 4HNE (**P**). Error bars: standard deviation of three independent trials Asterisk, *p* < 0.05. Statistical differences were calculated using ordinary ANOVA with Bonferroni correction. CRC, colorectal cancer; 5FU, 5-fluorouracil; IC, inhibitory concentration; TS, thymidylate synthase; DPD, dihydropyrimidine dehydrogenase; MTHFR, methylenetetrahydrofolate reductase; TYMP, thymidine phosphorylase; OXPHOS, oxidative phosphorylation; OCR, oxygen consumption rate; ECAR, extracellular acidification rate; MtVol, mitochondrial volume; MMP, mitochondrial membrane potential; TMRE, tetramethylrhodamine ethyl ester; DHR123, dihydrorhodamine 123; mtSOX, mitochondrial superoxide; 4HNE, 4-hydroxynonenal; ANOVA, analysis of variance.

The IC_50_ values of the HT29R and CT26R 5FU-resistant cell lines increased from 9.5 to 14.1 and from 7.6 to 10.8, respectively (Figure 4A). The proliferation of both resistant cell lines was reduced compared to those of the parent cell lines (Figure 4B). The expression levels of known 5FU resistance-related genes (*TS*, *DPD*, *MTHFR*, and *TYMP*) did not differ significantly between the parent and resistant cell lines (Figure 4C). Assessment of OXPHOS revealed reduced basal OCR, maximum OCR, and ATP in both resistant cell lines compared with the parent cell lines (Figure 4D–F). In contrast, glycolysis was enhanced in both resistant cell lines compared with the parent cell lines (Figure 4G). The energy metabolism profiles of both resistant cell lines showed glycolytic changes (Figure 4H). Mitochondrial volume and MMP were lower in both resistant cell lines compared with the parent cell lines (Figure 4I,J). In contrast, oxidative stress was decreased in both resistant cell lines compared with the parent cell lines (Figure 4K–M). Furthermore, oxidative stress upon 5FU treatment decreased in the resistant cell lines (Figure 4N–P).

**Figure 5:**
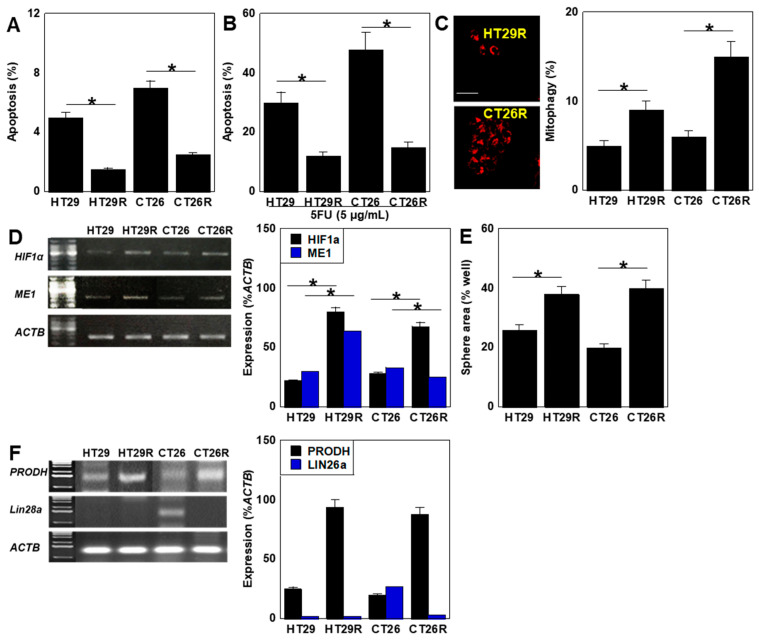
Stemness of 5FU-resistant cell lines derived from two CRC cell lines. 5FU-resistant HT29R and CT26R cell lines were established from HT29 and CT26 cells, respectively, by continuous treatment with low-dose 5FU (IC_5_) for 50 passages. (**A**) Apoptosis. (**B**) 5FU (5 μg/mL for 48 h). (**C**) Mitophagy. Scale bar: 50 μm. Right panel: semi-quantification of fluorescence images. (**D**) Expression of stemness-associated genes HIF1α and ME1. Right panel: semi-quantification of RT-PCR signals. (**E**) Sphere areas. (**F**) Expression of the naïve/prime transition-associated genes *PRODH* and *LIN28a*. Right panel: semi-quantification of RT-PCR signals. Error bars: standard deviation of three independent trials. Asterisk, *p* < 0.05. Statistical differences were calculated using ordinary ANOVA with Bonferroni correction. CRC, colorectal cancer; 5FU, 5-fluorouracil; ACTB, β-actin; RT-PCR, reverse transcription polymerase chain reaction; PRODH, proline dehydrogenase; HIF1A, hypoxia-inducible 1α; ME1, cytosolic NADPH dehydrogenase 1 (malic enzyme 1); ANOVA, analysis of variance.

Both resistant cell lines showed decreased apoptosis compared with the parent cell lines (Figure 5A). Moreover, 5FU-induced apoptosis was also reduced (Figure 5B). Mitophagy was also increased in both resistant cell lines compared with the parent cell lines (Figure 5C). Gene expression of *HIF1α* [35], which promotes the development of drug resistance in pancreatic cancer cell lines, and cytosolic NADPH dehydrogenase 1 (malic enzyme 1, *ME1*) [50,51], which promotes glutaminolysis and is related to stemness in oral cancer cells, were increased in HT29R and CT26R cells, whereas only *HIF1α* showed increased expression in CT26R cells (Figure 5D). The sphere formation ability was increased in both resistant cell lines (Figure 5E). Furthermore, examination of the expression levels of the naïve-like stem cell-associated marker *PRODH* and prime-like stem cell-associated marker *LIN28A* showed increased *PRODH* expression in both resistant cell lines, whereas the expression of *LIN28A* observed in CT26 cells was almost completely lost in CT26R cells (Figure 5F).

Thus, the resistant cells established from the two CRC cell lines showed enhanced glycolysis due to the reprogramming of energy metabolism and the promotion of naïve-like changes in stemness.

### 2.5. Effect of LAA on 5FU-Resistant CRC Cell Lines

**Figure 6:**
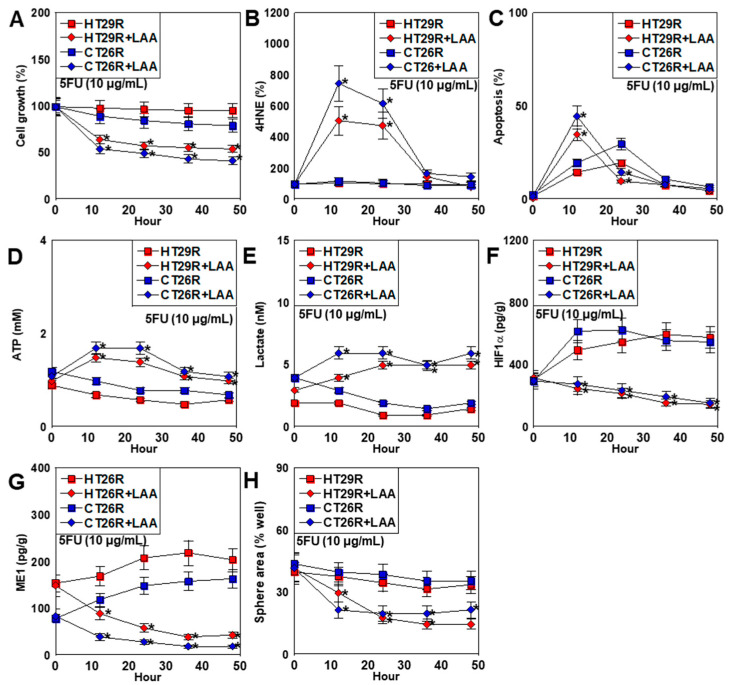
Effect of lauric acid (LAA) on stemness in 5FU-resistant CRC cell lines. The 5FU-resistant cell lines HT29R and CT26R were treated with 5FU (10 μg/mL) with or without LAA (40 μg/mL) for 40 h. (**A**) Cell growth. (**B**) 4HNE. (**C**) Apoptosis. (**D**) ATP. (**E**) Lactate levels in the culture medium. (**F**,**G**) Protein levels of HIF1α (**F**) and ME1 (**G**). (**H**) Sphere areas. Error bars: standard deviation of three independent trials. Asterisk, *p* < 0.05. Statistical differences were calculated using ordinary ANOVA with Bonferroni correction. CRC, colorectal cancer; 5FU, 5-fluorouracil; LAA, lauric acid; 4HNE, 4-hydroxynonenal; HIF1α, hypoxia inducible 1α; ME1, cytosolic NADPH dehydrogenase 1 (malic enzyme 1); ANOVA, analysis of variance.

We previously demonstrated the effectiveness of LAA against drug resistance in pancreatic cancer cell lines [35]. Therefore, we examined the effect of LAA on the two 5FU-resistant cell lines established in the current study (Figure 6). LAA significantly inhibited cell proliferation in resistant cells treated with 5FU equivalent to the IC_50_ (Figure 6A). No change was observed in oxidative stress for treatment with 5FU alone, whereas a high degree of induction was observed early in LAA co-treatment (Figure 6B). Apoptosis increased in the middle phase for treatment with 5FU alone, whereas it increased early in LAA co-treatment (Figure 6C). LAA co-treatment enhanced ATP production, which decreased in the late phase (Figure 6D). In contrast, LAA co-treatment enhanced lactate concentration in the culture medium, a marker of glycolysis (Figure 6E). Treatment with 5FU alone increased HIF1α and ME1 protein levels, whereas those levels were suppressed by simultaneous treatment with LAA (Figure 6F,G). Finally, the sphere formation ability showed an early decrease during simultaneous treatment with LAA (Figure 6H).

Thus, simultaneous treatment with 5FU and LAA can overcome 5FU resistance by enhancing oxidative stress through forced promotion of OXPHOS and suppression of the expression of stemness-associated proteins.

## 3. Discussion

In this study, we used two colon cancer cell lines, HT29 and CT26, to examine changes in stem cell properties and energy metabolism during the acquisition of 5FU resistance. Compared with CT26 cells, which are OXPHOS-dominant and energetic, HT29 cells show glycolytic and quiescent properties. However, upon acquiring 5FU resistance, both cell lines exhibited enhanced stemness and changed to a state of low proliferative ability with glycolytic dominance and reduced reactive oxygen species (ROS) production. In contrast, LAA reversed 5FU resistance by inducing OXPHOS, increasing ROS levels, and inducing cell death.

The relationship between CSCs and normal stem cells has attracted considerable research attention. Compared with tissue stem cells, which are almost quiescent, pluripotent stem cells (PSCs) exhibit vigorous proliferation [52,53]. Therefore, CSCs resemble PSCs [54]. Unlike somatic cells, PSCs possess a unique metabolic pathway highly dependent on glycolysis [55]. In this respect, CSCs that exhibit the Warburg effect resemble PSCs. The crista morphology also shows immature and fragmented mitochondria in PSCs [56,57], resembling CSCs that exhibit the Warburg effect [55,58]. Conversely, enhanced OXPHOS is associated with a loss of pluripotency [59] and was correlated with higher colonocyte differentiation in CT26 cells than in HT29 cells in the current study.

Pluripotency is currently recognized in two distinct states: naïve and primed. The naïve state corresponds to the pre-implantation state, while the prime state corresponds to the post-implantation state, which is more mature and has a degree of differentiation bias [60]. To examine the correspondence between these two states and CRC cells, we investigated the expression of *KLF4* [46] and *PRODH* [47] as markers of the naïve state and *LIN28A* [48] and *DNMT3B* [49] as markers of the prime state. We observed that HT29 cells predominantly expressed *KLF4* and *PRODH*, which suggests that HT29 cells exhibit a naïve-like phenotype. In contrast, CT26 cells predominantly expressed *LIN28A* and *DNMT3B* and exhibited a prime-like phenotype. These findings correlated with the proliferation, energy metabolism, ROS production, and differentiation of both CRC cell lines. In contrast, after acquiring 5FU resistance, both CRC cell lines showed increased expression of naïve markers and decreased proliferation, MMP, and ROS production, suggesting a change to a naïve-like state. In particular, Lin28a expression was lost in CT26R cells, indicating that the stem cell phenotype changed from primed to naïve, suggesting that this may be due to reprogramming of energy metabolism. In CSCs, low MMP levels reduce mitochondrial ROS levels [61]. Thus, naïve-like stemness and changes in energy metabolism may be associated with 5FU resistance.

In this study, *HIF1α* and *ME1* expression levels were higher in the 5FU-resistant cell lines. Although our experimental system was not hypoxic, increased ROS levels stabilized the HIF1α protein [62]. This suggests that 5FU-induced ROS production activated HIF1α in the 5FU-resistant cells. HIF1α increases dynamin-related protein 1-dependent mitochondrial fission and promotes the Warburg phenotype [63,64,65,66]. Hypoxia, which induces HIF1α, also causes resistance to anticancer drugs through HIF1α-dependent mitochondrial changes [67] and HIF1α-induced glycolytic flux [52,53,68]. Fission mitochondria are present in cells that do not require high respiratory activity [69]. HIF1α also suppresses mitochondrial biogenesis and PTEN-induced kinase 1 transcription [70]. Moreover, we previously showed that stabilization of HIF1α promotes energy metabolism reprogramming and induces early gemcitabine resistance [35].

ME1 directly converts the malate produced in the tricarboxylic acid (TCA) cycle to pyruvate, while increased pyruvate levels promote glutaminolysis [71]. Glutaminolysis is a metabolic phenotype associated with CSCs, especially those with *KRAS* mutations [72], and has attracted attention as a potential therapeutic target [73,74]. Glutaminolysis promotes stemness by activating Yes-associated proteins, transcriptional coactivators with PDZ-binding motifs, and WNT [72,75]. We previously demonstrated the correlation between increased ME1 expression and malignancy in oral squamous cell carcinomas, particularly invasiveness due to epithelial–mesenchymal transition at the cancer front [50,51].

In the current study, LAA promoted OXPHOS in the resistant cell lines, accompanied by increased mitochondrial ROS. This effect promotes apoptosis and may explain the antitumor effects of LAA. Similar results were reported in previous studies [36,37]. However, LAA does not increase mitochondrial ROS levels in normal cells [76,77,78]. Thus, the effects in cancer cells may be attributed to mitochondrial disorders in these cells [79,80,81]. Mitochondrial gene mutations are frequently observed in cancer and cancer cells by altering mitochondrial DNA copy number, energy metabolism, oxidative stress, nuclear–mitochondrial interactions, and the cancer microenvironment [82,83,84]. This may contribute to carcinogenicity [85]. Therefore, LAA may have different thresholds of cytotoxicity between cancer and normal cells and could be used as a cancer treatment that distinguishes between normal and cancer tissues.

In this study, we focused on a small molecule called LAA and clarified its antitumor effect, especially its ability to suppress anticancer drug resistance. In addition, small molecule compounds that are expected to overcome anticancer drug resistance are attracting attention. Thiocolchicoside has a muscle relaxant effect through inhibition of nicotinic acetylcholine receptors and GABA. Like LAA, thiocolchicoside also enhances skin permeability and improves drug delivery [86]. Salinomycin, an antibiotic, suppresses cancer stemness through autophagy and enhances drug sensitivity [87]. Beta-elemene is an extract of Curcuma wenyujin that inhibits the ATP-binding cassette subfamily B member 1 transporter and induces apoptosis and autophagy through the suppression of exosome transmission, promoting drug sensitivity [88]. Metformin, a type 2 diabetes drug, is expected to contribute to overcoming anticancer drug resistance by inducing apoptosis and ferroptosis in cancer cells through regulation of AMPK activity and effects on the immune system [89,90]. In addition, berberine, a Berberis extract, is expected to be effective against drug resistance because it suppresses cancer stem cell activity [91]. In addition, pterostilbene, a blueberry component, is expected to be effective against drug resistance because it promotes cell death in cancer stem cells through mitochondrial iron accumulation [92,93,94]. By combining such small molecule compounds with existing chemotherapy or molecular targeted drugs, it is expected to promote drug efficacy, suppress the acquisition of resistance, and reduce side effects. The results of this study demonstrated that CRCs develop resistance to 5FU due to changes in stemness accompanied by the reprogramming of energy metabolism, similar to the development of gemcitabine resistance in pancreatic cancer cell lines. Moreover, we showed that LAA is effective against developing 5FU resistance. However, the molecular mechanisms underlying these alterations in stemness have not yet been fully elucidated. Additional studies are needed to elucidate this mechanism and develop strategies for preventing resistance to anticancer drugs. In addition, in this and our previous studies, we investigated key anticancer drugs used in chemotherapy: gemcitabine for pancreatic cancers and 5FU for CRCs. Further studies are needed to examine the role of alterations in stemness and reprogramming of energy metabolism in the mechanism of resistance acquisition for other anticancer drugs used in various tumors and whether LAA is effective in overcoming these other drug resistances. Ultimately, its efficacy must be established in humans through therapeutic experiments in animal models as well as clinical studies.

## 4. Materials and Methods

### 4.1. Cell Lines

HT29 human CRC cell lines were purchased from Dainihon Pharmacy Co. (Tokyo, Japan). The CT26 murine CRC cell line was a gift from Professor I. J. Fidler (MD Anderson Cancer Center, Houston, TX, USA). The cells were cultured in Dulbecco’s modified Eagle’s medium (DMEM) supplemented with 10% fetal bovine serum at 37 °C in 5% CO_2_. The cells were treated with 5FU (Sigma-Aldrich, St. Louis, MO, USA) and/or LAA (40 μg/mL, Wako Pure Chemical Corporation, Osaka, Japan). The 5FU-resistant cell lines were derived from HT29 and CT26 cells by continuous low-dose 5FU treatment (IC_5_) for 50 passages. Apoptosis was assessed via the examination of 1000 cells, which were stained with Hoechst 33342 dye (Life Technologies, Carlsbad, CA, USA) and viewed using a fluorescent microscope.

### 4.2. [3-(4,5-Dimethylthiazol-2-yl)-5-(3-carboxymethoxyphenyl)-2-(4-sulfophenyl)-2H-tetrazolium] (MTS) Assay

MTS assays were performed using a CellTiter 96 aqueous one-solution cell proliferation assay kit (Promega Biosciences Inc., San Luis Obispo, CA, USA). The plates were read using a Multiskan FC (Thermo Fisher Scientific, Waltham, MA, USA) microplate photometer at a wavelength of 490 nm. The MTS value of cells cultured with the control oligonucleotide was used as the control.

### 4.3. Mitochondrial Imaging

Mitochondrial function was examined using fluorescent probes. After treatment with or without LAA (40 μg/mL), the cells were incubated with the probes for 30 min at 37 °C and then photographed using an All-in-One fluorescence microscope (KEYENCE, Osaka, Japan). We used dihydrorhodamine 123 (DHR123) (100 μM, Dojindo, Kumamoto, Japan) to measure mitochondrial H_2_O_2_, MitoSOX (mitochondrial superoxide) (10 μM, AAT Bioquest Inc., Sunnyvale, CA, USA) to assess oxidative stress, MitoGreen (100 nM, PromoCell GmbH, Heidelberg, Germany) to assess mitochondrial volume, and tetramethylrhodamine ethyl ester (TMRE) (200 nM, Sigma-Aldrich) to assess mitochondrial membrane potential. Mitophagy was detected using the Mitophagy Detection Kit (Dojindo) according to the manufacturer’s instructions.

### 4.4. Protein Extraction

Whole-cell lysates were prepared as previously described using radioimmunoprecipitation assay buffer containing 0.1% sodium dodecyl sulfate (Thermo Fisher Scientific, Tokyo, Japan) [95]. Protein assays were performed using the Protein Assay Rapid Kit (Wako).

### 4.5. Enzyme-Linked Immunosorbent Assay (ELISA) and Fluorometric Assay

We used an ELISA kit to measure the concentrations of 4HNE, lactate, ALP, MUC2, ATP, HIF1α, and ME1 in whole-cell lysates (Table 1). The assay was performed according to the manufacturer’s instructions.

### 4.6. Sphere Assay

Cells (1000 cells/well) were seeded on uncoated bacteriological 35 mm dishes (Corning Inc., Corning, NY, USA) with 3D Tumorsphere Medium XF (Sigma-Aldrich) and cultured with or without LAA (40 μg/mL). After seven days, sphere images were captured using a computer and the sphere numbers were measured using NIH ImageJ software (version 1.52, Bethesda, MD, USA).

### 4.7. Mitochondrial Stress Test

To analyze mitochondrial respiration and ATP production, we used a Seahorse XF Analyzer (Agilent Technologies, Santa Clara, CA, USA) to measure extracellular flux in live cells. The cells were collected immediately after LAA treatment (40 µg/mL, 48 h), transferred to an XF plate at densities of 2 × 10^4^ cells/well, and incubated overnight. The following day, the medium on the XF plate was replaced with XF DMEM, 1 h before the assay. Mito Stress Test (Seahorse XF Cell Mito Stress Test, Agilent Technologies) was performed according to the manufacturer’s protocol. The OCR was measured under the following conditions: 2 µM oligomycin, 0.5 µM carbonyl cyanide-p-trifluoromethoxyphenylhydrazone, and 0.5 µM rotenone/antimycin A. The OCR was normalized to the total cellular protein concentration, which was determined after protein extraction from the analyzed cells.

### 4.8. Glycolytic Stress Test

The extracellular acidification rate (ECAR) of CRC cells was measured using a modified glycolytic stress test on a Seahorse XFe24 Extracellular Flux Analyzer instrument with Seahorse XF24 FluxPaks (Agilent Technologies, Santa Clara, CA, USA). CRC cells were cultured in growth medium in six-well plates with ascites or culture medium before the Seahorse experiments. CRC cells (1 × 10^4^ cells/well) were plated in XF base medium (Agilent Technologies, Santa Clara, CA, USA) containing 200 mM L-glutamine and 5 mM HEPES, as recommended by the manufacturer for glycolytic assays. The sensor cartridge apparatus was rehydrated one day in advance by adding 1 mL XF Calibrant to each well and incubating at 37 °C until needed. The injection ports of the sensor cartridge apparatus were then loaded with the following drugs, in chronological order of four injections, to meet the indicated final concentrations in the wells: 10 mM glucose, 1 µM oligomycin, 1 µM rotenone and 5 µM antimycin A (combined injection), and 50 mM 2-deoxyglucose. Treatment with the rotenone/antimycin combination allowed the assessment of the impact of electron transport on ECAR by respiratory acidification coupled with the passage of some glycolytic pyruvate through the TCA cycle to supply respiration.

### 4.9. Reverse Transcription Polymerase Chain Reaction (RT-PCR)

To assess human and murine mRNA expression, RT-PCR was performed using 0.5 µg of total RNA extracted from the three cell lines using an RNeasy kit (Qiagen, Germantown, MD, USA). The primer sets are listed in Table 1 and were synthesized by Sigma Genosys (Ishikari, Japan). The PCR products were electrophoresed on a 2% agarose gel and stained with ethidium bromide. β-Actin mRNA was amplified and used as the internal control.

### 4.10. Statistical Analysis

Statistical significance was calculated using a two-tailed Fisher exact test, ordinary analysis of variance, and InStat software version 3.1 (GraphPad, San Diego, CA, USA). Two-sided *p* < 0.05 was considered statistically significant.

## Figures and Tables

**Table 1:** RT-PCR primers and ELISA kits.

RT-PCR Primers				
Gene	Species	ID	Left	Right
*Actb*	Mouse	NM_007393.5	AGCCATGTACGTAGCCATCC	CTCTCAGCTGTGGTGGTGAA
*ACTB*	Human	NM_001101.3	GGACTTCGAGCAAGAGATGG	AGCACTGTGTTGGCGTACAG
*Lgr5*	Mouse	NM_010195.2	CATTCACTTTTGGCCGTTTT	AGGGCCAACAGGACACATAG
*LGR5*	Human	AF061444.1	CTCTTCCTCAAACCGTCTGC	GATCGGAGGCTAAGCAACTG
*Klf4*	Mouse	NM_010637.3	CTGAACAGCAGGGACTGTCA	GTGTGGGTGGCTGTTCTTTT
*KLF4*	Human	KJ901962.1	CCCACACAGGTGAGAAACCT	CCCACACAGGTGAGAAACCT
*Ns*	Mouse	BC037996.1	ATGTGGGGAAAAGCAGTGTC	TGGGGGAGTTACAAGGTGAG
*NS*	Human	BC001024.2	ATTGCCAACAGTGGTGTTCA	AATGGCTTTGCTGCAAGTTT
*Lin28a*	Mouse	NM_145833.1	GTGTCCAACCAGCAGTTTGC	CTCTTCCTCTTCCTCCCGGA
*LIN28A*	Human	NM_024674.6	CGTGTCCAACCAGCAGTTTG	TGGCTTTCCCTGTGCACTAG
*Prodh*	Mouse	AF120279.1	AAGCAGTATCAGGTGCACCC	CCTCCTCAGTGAACCGTGAC
*PRODH*	Human	AF120278.1	GGTAGAGTCAGCGATGACGG	TGTGTTGAAGATGAGCGGCT
*Dnmt3b*	Mouse	BC105677.1	TGTGGGGAAAGATCAAGGGC	CGTTCTCGGCTCTCCTCATC
*DNMT3B*	Human	AF331857.1	TTCTCCGAGGTCTCTGCAGA	CTGCCACAAGACAAACAGCC
*Hif1a*	Mouse	AF003695.1	TGCTTGCCAAAAGAGGTGGA	CAGAAGGACTTGCTGGCTGA
*HIF1A*	Human	AF208487.1	GAAAGCGCAAGTCCTCAAAG	TGGGTAGGAGATGGAGATGC
*Me1*	Mouse	NM_008615.2	GGAGTTGCTGCAATTGGTGG	TGCAGGCCACGGATAACAAT
*ME1*	Human	NM_002395.5	GGATTGCACACCTGATTGTG	TCTTCATGTTCATGGGCAAA
*Ts*	Mouse	NM_021288.4	TTCAAGAAGGAGGACCGCAC	CACGCCCAGACCCATATCTC
*TS*	Human	NM_001071.4	CTGGGGCAGATCCAACACAT	CTGGCGATGTTGAAAGGCAC
*Dpd*	Mouse	NM_170778.3	GTATGGCCCTGGACAAAGCT	GCAGTTCCTGACACTCCTCC
*DPD*	Human	NM_000110.4	GTATGGCCCTGGACAAAGCT	GCAATGGAGGTCACAGCTCT
*Mthfr*	Mouse	NM_001161798.1	CAGCTGGGCACTGTTATCCA	GCTTCCCAGTGGTCACCTAC
*MTHFR*	Human	NM_001330358.2	TCTACCGTACCCAGGAGTGG	GTGGGCTGGATGATCTCTCG
*Tymp*	Mouse	NM_138302.3	CTGGAGGTGGAAGAAGCGTT	GGGAGGACAAGTTCAGCGAA
*TYMP*	Human	BC018160.1	CAAGGTGCCAATGATCAGCG	CAGGTCCCTTAAGTCTGGCG
ELISA				
Target	Species	Cat#	Company	
Alp	Mouse	ab285274	Abcam, Waltham, MA, USA	
ALP	Human	ab285149	Abcam, Waltham, MA, USA	
Muc2	Mouse	M0EB0548	AssayGenie, Dublin, Ireland	
MUC2	Human	ab282871	Abcam, Waltham, MA, USA	
4HNE	-	ab287803	Abcam, Waltham, MA, USA	
Lactate	-	ab65331	Abcam, Waltham, MA, USA	
ATP	-	ab83355	Abcam, Waltham, MA, USA	
Hif1α	Mouse	#88-8022-88	Thermo Fisher, Tokyo, Japan	
HIF1α	Human	#A43658	Thermo Fisher, Tokyo, Japan	
Me1	Mouse	E1556Mo	Bioassay Technology Laboratory, Shanghai, China	
ME1	Human	ABIN6231269	Antibodies-online.com, Limerick, PA, USA	

RT-PCR, reverse transcription polymerase chain reaction; ELISA, enzyme-linked immunosorbent assay; ACTB, β-actin; LGR5, leucine-rich repeat-containing G-protein coupled receptor 5; KLF4, Krüppel-like factor 4; NS, nucleostemin; PRODH, proline dehydrogenase; 4HNE, 4-hyrdoxynonenal; Dnmt3b, DNA methyl transferase 3B; HIF1A, hypoxia-inducible factor 1-alpha; ME1, cytosolic NADPH dehydrogenase 1 (malic enzyme 1); ALP, alkaline phosphatase; MUC2, mucin 2; ATP, adenine triphosphate; TS, thymidylate synthase; DPD, dihydropyrimidine dehydrogenase; MTHFR, methylenetetrahydrofolate reductase; TYMP, thymidine phosphorylase.

## Data Availability

The original contributions presented in this study are included in the article. Further inquiries can be directed to the corresponding authors.

## Abbreviations

5FU	5-fluorouracil
CRC	colorectal cancer
OXPHOS	oxidative phosphorylation
ROS	reactive oxygen species
CSC	cancer stem cell
TS	thymidylate synthase
DPD	dihydropyrimidine dehydrogenase
MTHFR	methylenetetrahydrofolate reductase
TYMP	thymidine phosphorylase
HIF1α	hypoxia-inducible factor-1α
LAA	lauric acid
MCFA	medium-chain fatty acid
ALP	alkaline phosphatase
MUC2	mucin 2
MMP	mitochondrial membrane potential
4HNE	4-hydroxynonenal
LGR5	leucine-rich repeat-containing G-protein coupled receptor 5
NS	Nucleostemin
KLF4	Krüppel-like factor 4
PRODH	proline dehydrogenase
Dnmt3b	DNA methyltransferase-3b
ME1	cytosolic NADPH dehydrogenase 1 (malic enzyme 1)
PSC	pluripotent stem cell

## References

[1] Li J., Yang B., Teng Z., Liu Y., Li D., Qu X. (2024). Efficacy and safety of first-line treatments for advanced hepatocellular carcinoma patients: A systematic review and network meta-analysis. Front. Immunol..

[2] Orillard E., Adhikari A., Malouf R.S., Calais F., Marchal C., Westeel V. (2024). Immune checkpoint inhibitors plus platinum-based chemotherapy compared to platinum-based chemotherapy with or without bevacizumab for first-line treatment of older people with advanced non-small cell lung cancer. Cochrane Database Syst. Rev..

[3] Matsuoka T., Yashiro M. (2024). Molecular Mechanism for Malignant Progression of Gastric Cancer Within the Tumor Microenvironment. Int. J. Mol. Sci..

[4] Mustafa M., Rashed M., Winum J.Y. (2024). Novel Anticancer Drug Discovery Strategies Targeting Hypoxia-Inducible factors. Expert Opin. Drug Discov..

[5] Rastegar-Pouyani N., Abdolvahab M.H., Farzin M.A., Zare H., Kesharwani P., Sahebkar A. (2024). Targeting cancer-associated fibroblasts with pirfenidone: A novel approach for cancer therapy. Tissue Cell.

[6] Mittal V., El Rayes T., Narula N., McGraw T.E., Altorki N.K., Barcellos-Hoff M.H. (2016). The Microenvironment of Lung Cancer and Therapeutic Implications. Adv. Exp. Med. Biol..

[7] Yoo H., Kim Y., Kim J., Cho H., Kim K. (2024). Overcoming Cancer Drug Resistance with Nanoparticle Strategies for Key Protein Inhibition. Molecules.

[8] Kirtane A.R., Kalscheuer S.M., Panyam J. (2013). Exploiting nanotechnology to overcome tumor drug resistance: Challenges and opportunities. Adv. Drug Deliv. Rev..

[9] Liu Z., Parveen N., Rehman U., Aziz A., Sheikh A., Abourehab M.A.S., Guo W., Huang J., Wang Z., Kesharwani P. (2023). Unravelling the enigma of siRNA and aptamer mediated therapies against pancreatic cancer. Mol. Cancer.

[10] Niharika, Garg M. (2024). Understanding the autophagic functions in cancer stem cell maintenance and therapy resistance. Expert Rev. Mol. Med..

[11] Jamroze A., Liu X., Tang D.G. (2024). Treatment-induced stemness and lineage plasticity in driving prostate cancer therapy resistance. Cancer Heterog. Plast..

[12] Chen L., Zhang H., Shang C., Hong Y. (2024). The Role and Applied Value of Mitochondria in Glioma-Related Research. CNS Neurosci. Ther..

[13] Hu Y., Liu W., Fang W., Dong Y., Zhang H., Luo Q. (2024). Tumor energy metabolism: Implications for therapeutic targets. Mol. Biomed..

[14] Li J., Zhang W., Chen L., Wang X., Liu J., Huang Y., Qi H., Wang T., Li Q. (2024). Targeting extracellular matrix interaction in gastrointestinal cancer: Immune modulation, metabolic reprogramming, and therapeutic strategies. Biochim. Biophys. Acta Rev. Cancer.

[15] Monzer A., Ghamlouche F., Wakimian K., Ballout F., Al Bitar S., Yehya A., Kanso M., Saheb N., Tawil A., Doughan S. (2024). ONC206, an imipridone derivative, demonstrates anti-colorectal cancer activity against stem/progenitor cells in 3D cell cultures and in patient-derived organoids. Pharmacol. Rep..

[16] Di C., Zhao Y. (2015). Multiple drug resistance due to resistance to stem cells and stem cell treatment progress in cancer (Review). Exp. Ther. Med..

[17] Saha T., Lukong K.E. (2022). Breast Cancer Stem-Like Cells in Drug Resistance: A Review of Mechanisms and Novel Therapeutic Strategies to Overcome Drug Resistance. Front. Oncol..

[18] Warburg O., Wind F., Negelein E. (1927). The Metabolism of tumors in the body. J. Gen. Physiol..

[19] Cordani M., Michetti F., Zarrabi A., Zarepour A., Rumio C., Strippoli R., Marcucci F. (2024). The role of glycolysis in tumorigenesis: From biological aspects to therapeutic opportunities. Neoplasia.

[20] Sancho P., Burgos-Ramos E., Tavera A., Bou Kheir T., Jagust P., Schoenhals M., Barneda D., Sellers K., Campos-Olivas R., Graña O. (2015). MYC/PGC-1α Balance Determines the Metabolic Phenotype and Plasticity of Pancreatic Cancer Stem Cells. Cell Metab..

[21] Elhinnawi M.A., Boushra M.I., Hussien D.M., Hussein F.H., Abdelmawgood I.A. (2024). Mitochondria’s Role in the Maintenance of Cancer Stem Cells in Hepatocellular Carcinoma. Stem Cell Rev. Rep..

[22] Uslu C., Kapan E., Lyakhovich A. (2024). Cancer resistance and metastasis are maintained through oxidative phosphorylation. Cancer Lett..

[23] Friess D., Brauer S., Pöysti A., Choudhury C., Harris L. (2024). Tools to study neural and glioma stem cell quiescence. Trends Neurosci..

[24] Du Y., Gupta P., Qin S., Sieber M. (2023). The role of metabolism in cellular quiescence. J. Cell. Sci..

[25] McQuade R.M., Stojanovska V., Bornstein J.C., Nurgali K. (2017). Colorectal Cancer Chemotherapy: The Evolution of Treatment and New Approaches. Curr. Med. Chem..

[26] Vodenkova S., Buchler T., Cervena K., Veskrnova V., Vodicka P., Vymetalkova V. (2020). 5-fluorouracil and other fluoropyrimidines in colorectal cancer: Past, present and future. Pharmacol. Ther..

[27] Gmeiner W.H., Okechukwu C.C. (2023). Review of 5-FU resistance mechanisms in colorectal cancer: Clinical significance of attenuated on-target effects. Cancer Drug Resist..

[28] Kumar A., Singh A.K., Singh H., Thareja S., Kumar P. (2022). Regulation of thymidylate synthase: An approach to overcome 5-FU resistance in colorectal cancer. Med. Oncol..

[29] Zhang Y.H., Luo D.D., Wan S.B., Qu X.J. (2020). S1PR2 inhibitors potently reverse 5-FU resistance by downregulating DPD expression in colorectal cancer. Pharmacol. Res..

[30] Xie P., Mo J.L., Liu J.H., Li X., Tan L.M., Zhang W., Zhou H.H., Liu Z.Q. (2020). Pharmacogenomics of 5-fluorouracil in colorectal cancer: Review and update. Cell. Oncol..

[31] Ibrahim S., Li G., Hu F., Hou Z., Chen Q., Luo X., Hu J., Feng Y. (2018). PIK3R3 promotes chemotherapeutic sensitivity of colorectal cancer through PIK3R3/NF-kB/TP pathway. Cancer Biol. Ther..

[32] Baba H., Watanabe M., Okabe H., Miyamoto Y., Sakamoto Y., Baba Y., Iwatsuki M., Chikamoto A., Beppu T. (2012). Upregulation of ERCC1 and DPD expressions after oxaliplatin-based first-line chemotherapy for metastatic colorectal cancer. Br. J. Cancer.

[33] Sreekumar R., Al-Saihati H., Emaduddin M., Moutasim K., Mellone M., Patel A., Kilic S., Cetin M., Erdemir S., Navio M.S. (2021). The ZEB2-dependent EMT transcriptional programme drives therapy resistance by activating nucleotide excision repair genes ERCC1 and ERCC4 in colorectal cancer. Mol. Oncol..

[34] Fujiwara-Tani R., Sasaki T., Bhawal U.K., Mori S., Ogata R., Sasaki R., Ikemoto A., Kishi S., Fujii K., Ohmori H. (2024). Nuclear MAST4 Suppresses FOXO3 through Interaction with AKT3 and Induces Chemoresistance in Pancreatic Ductal Carcinoma. Int. J. Mol. Sci..

[35] Takagi T., Fujiwara-Tani R., Mori S., Kishi S., Nishiguchi Y., Sasaki T., Ogata R., Ikemoto A., Sasaki R., Ohmori H. (2023). Lauric Acid Overcomes Hypoxia-Induced Gemcitabine Chemoresistance in Pancreatic Ductal Adenocarcinoma. Int. J. Mol. Sci..

[36] Scheig R. (1968). Absoption of dietary fat: Use of medium-chain triglycerides in malabsorption. Am. J. Clin. Nutr..

[37] Hagenfeldt L., Wahren J., Pernow B., Räf L. (1972). Uptake of individual free fatty acids by skeletal muscle and liver in man. J. Clin. Investig..

[38] Papamandjaris A.A., MacDougall D.E., Jones P.J. (1998). Medium chain fatty acid metabolism and energy expenditure: Obesity treatment implications. Life Sci.

[39] Fauser J.K., Matthews G.M., Cummins A.G., Howarth G.S. (2013). Induction of apoptosis by the medium-chain length fatty acid lauric acid in colon cancer cells due to induction of oxidative stress. Chemotherapy.

[40] Metges C.C., Wolfram G. (1991). Medium- and long-chain triglycerides labeled with 13C: A comparison of oxidation after oral or parenteral administration in humans. J. Nutr..

[41] Kadochi Y., Mori S., Fujiwara-Tani R., Luo Y., Nishiguchi Y., Kishi S., Fujii K., Ohmori H., Kuniyasu H. (2017). Remodeling of energy metabolism by a ketone body and medium-chain fatty acid suppressed the proliferation of CT26 mouse colon cancer cells. Oncol. Lett..

[42] Ogata R., Mori S., Ohmori H., Kishi S., Fujiwara-Tani R., Sasaki T., Nishiguchi Y., Nakashima C., Goto K., Kawahara I. (2022). Suppressive GLI2 fragment enhances liver metastasis in colorectal cancer. Oncotarget.

[43] Kita M., Fujiwara-Tani R., Kishi S., Mori S., Ohmori H., Nakashima C., Goto K., Sasaki T., Fujii K., Kawahara I. (2023). Role of creatine shuttle in colorectal cancer cells. Oncotarget.

[44] Du P., Wu J. (2024). Hallmarks of totipotent and pluripotent stem cell states. Cell Stem Cell.

[45] Varzideh F., Gambardella J., Kansakar U., Jankauskas S.S., Santulli G. (2023). Molecular Mechanisms Underlying Pluripotency and Self-Renewal of Embryonic Stem Cells. Int. J. Mol. Sci..

[46] Yoneyama Y., Zhang R.R., Kimura M., Cai Y., Adam M., Parameswaran S., Masaki H., Mizuno N., Bhadury J., Maezawa S. (2024). Inter-cellular mRNA Transfer Alters Human Pluripotent Stem Cell State. bioRxiv.

[47] Chen C., Liu Q., Chen W., Gong Z., Kang B., Sui M., Huang L., Wang Y.J. (2024). PRODH safeguards human naive pluripotency by limiting mitochondrial oxidative phosphorylation and reactive oxygen species production. EMBO Rep..

[48] Chen B., Jia M., Zhao G., Liu Y., Song Y., Sun M., Chi W., Wang X., Jiang Q., Xin G. (2024). STAG2 promotes naive-primed transition via activating Lin28a transcription in mouse embryonic stem cells. J. Biol. Chem..

[49] Zhao B., Yu X., Shi J., Ma S., Li S., Shi H., Xia S., Ye Y., Zhang Y., Du Y. (2024). A stepwise mode of TGFβ-SMAD signaling and DNA methylation regulates naïve-to-primed pluripotency and differentiation. Nat. Commun..

[50] Nakashima C., Yamamoto K., Fujiwara-Tani R., Luo Y., Matsushima S., Fujii K., Ohmori H., Sasahira T., Sasaki T., Kitadai Y. (2018). Expression of cytosolic malic enzyme (ME1) is associated with disease progression in human oral squamous cell carcinoma. Cancer Sci..

[51] Nakashima C., Kirita T., Yamamoto K., Mori S., Luo Y., Sasaki T., Fujii K., Ohmori H., Kawahara I., Mori T. (2020). Malic Enzyme 1 Is Associated with Tumor Budding in Oral Squamous Cell Carcinomas. Int. J. Mol. Sci..

[52] Ishihara N., Nomura M., Jofuku A., Kato H., Suzuki S.O., Masuda K., Otera H., Nakanishi Y., Nonaka I., Goto Y. (2009). Mitochondrial fission factor Drp1 is essential for embryonic development and synapse formation in mice. Nat. Cell Biol..

[53] Wakabayashi J., Zhang Z., Wakabayashi N., Tamura Y., Fukaya M., Kensler T.W., Iijima M., Sesaki H. (2009). The dynamin-related GTPase Drp1 is required for embryonic and brain development in mice. J. Cell Biol..

[54] Abdel-Haleem A.M., Lewis N.E., Jamshidi N., Mineta K., Gao X., Gojobori T. (2017). The Emerging Facets of Non-Cancerous Warburg Effect. Front. Endocrinol..

[55] Folmes C.D., Nelson T.J., Martinez-Fernandez A., Arrell D.K., Lindor J.Z., Dzeja P.P., Ikeda Y., Perez-Terzic C., Terzic A. (2011). Somatic oxidative bioenergetics transitions into pluripotency-dependent glycolysis to facilitate nuclear reprogramming. Cell Metab..

[56] Kawano I., Bazila B., Ježek P., Dlasková A. (2023). Mitochondrial Dynamics and Cristae Shape Changes During Metabolic Reprogramming. Antioxid. Redox Signal.

[57] Kondadi A.K., Anand R., Reichert A.S. (2020). Cristae Membrane Dynamics—A Paradigm Change. Trends Cell Biol..

[58] Chung S., Dzeja P.P., Faustino R.S., Perez-Terzic C., Behfar A., Terzic A. (2007). Mitochondrial oxidative metabolism is required for the cardiac differentiation of stem cells. Nat. Clin. Pract. Cardiovasc. Med..

[59] Varum S., Rodrigues A.S., Moura M.B., Momcilovic O., Easley C.A.t., Ramalho-Santos J., Van Houten B., Schatten G. (2011). Energy metabolism in human pluripotent stem cells and their differentiated counterparts. PLoS ONE.

[60] Nichols J., Smith A. (2009). Naive and primed pluripotent states. Cell Stem Cell.

[61] Zhang B.B., Wang D.G., Guo F.F., Xuan C. (2015). Mitochondrial membrane potential and reactive oxygen species in cancer stem cells. Fam. Cancer.

[62] Wu Z., Zuo M., Zeng L., Cui K., Liu B., Yan C., Chen L., Dong J., Shangguan F., Hu W. (2021). OMA1 reprograms metabolism under hypoxia to promote colorectal cancer development. EMBO Rep..

[63] Marsboom G., Toth P.T., Ryan J.J., Hong Z., Wu X., Fang Y.H., Thenappan T., Piao L., Zhang H.J., Pogoriler J. (2012). Dynamin-related protein 1-mediated mitochondrial mitotic fission permits hyperproliferation of vascular smooth muscle cells and offers a novel therapeutic target in pulmonary hypertension. Circ. Res..

[64] Elstrom R.L., Bauer D.E., Buzzai M., Karnauskas R., Harris M.H., Plas D.R., Zhuang H., Cinalli R.M., Alavi A., Rudin C.M. (2004). Akt stimulates aerobic glycolysis in cancer cells. Cancer Res..

[65] Mauro C., Leow S.C., Anso E., Rocha S., Thotakura A.K., Tornatore L., Moretti M., De Smaele E., Beg A.A., Tergaonkar V. (2011). NF-κB controls energy homeostasis and metabolic adaptation by upregulating mitochondrial respiration. Nat. Cell Biol..

[66] Papandreou I., Cairns R.A., Fontana L., Lim A.L., Denko N.C. (2006). HIF-1 mediates adaptation to hypoxia by actively downregulating mitochondrial oxygen consumption. Cell Metab..

[67] Chiche J., Rouleau M., Gounon P., Brahimi-Horn M.C., Pouysségur J., Mazure N.M. (2010). Hypoxic enlarged mitochondria protect cancer cells from apoptotic stimuli. J. Cell. Physiol..

[68] Plecitá-Hlavatá L., Engstová H., Alán L., Špaček T., Dlasková A., Smolková K., Špačková J., Tauber J., Strádalová V., Malínský J. (2016). Hypoxic HepG2 cell adaptation decreases ATP synthase dimers and ATP production in inflated cristae by mitofilin down-regulation concomitant to MICOS clustering. FASEB J..

[69] Westermann B. (2012). Bioenergetic role of mitochondrial fusion and fission. Biochim. Biophys. Acta.

[70] Kung-Chun Chiu D., Pui-Wah Tse A., Law C.T., Ming-Jing Xu I., Lee D., Chen M., Kit-Ho Lai R., Wai-Hin Yuen V., Wing-Sum Cheu J., Wai-Hung Ho D. (2019). Hypoxia regulates the mitochondrial activity of hepatocellular carcinoma cells through HIF/HEY1/PINK1 pathway. Cell Death Dis..

[71] Gdynia G., Sauer S.W., Kopitz J., Fuchs D., Duglova K., Ruppert T., Miller M., Pahl J., Cerwenka A., Enders M. (2016). The HMGB1 protein induces a metabolic type of tumour cell death by blocking aerobic respiration. Nat. Commun..

[72] Wong C.C., Xu J., Bian X., Wu J.L., Kang W., Qian Y., Li W., Chen H., Gou H., Liu D. (2020). In Colorectal Cancer Cells with Mutant KRAS, SLC25A22-Mediated Glutaminolysis Reduces DNA Demethylation to Increase WNT Signaling, Stemness, and Drug Resistance. Gastroenterology.

[73] Deshmukh A., Deshpande K., Arfuso F., Newsholme P., Dharmarajan A. (2016). Cancer stem cell metabolism: A potential target for cancer therapy. Mol. Cancer.

[74] Kao T.W., Chuang Y.C., Lee H.L., Kuo C.C., Shen Y.A. (2022). Therapeutic Targeting of Glutaminolysis as a Novel Strategy to Combat Cancer Stem Cells. Int. J. Mol. Sci..

[75] Koo J.H., Guan K.L. (2018). Interplay between YAP/TAZ and Metabolism. Cell Metab..

[76] Mori T., Ohmori H., Luo Y., Mori S., Miyagawa Y., Nukaga S., Goto K., Fujiwara-Tani R., Kishi S., Sasaki T. (2019). Giving combined medium-chain fatty acids and glucose protects against cancer-associated skeletal muscle atrophy. Cancer Sci..

[77] Nukaga S., Mori T., Miyagawa Y., Fujiwara-Tani R., Sasaki T., Fujii K., Mori S., Goto K., Kishi S., Nakashima C. (2020). Combined administration of lauric acid and glucose improved cancer-derived cardiac atrophy in a mouse cachexia model. Cancer Sci..

[78] Tham Y.Y., Choo Q.C., Muhammad T.S.T., Chew C.H. (2020). Lauric acid alleviates insulin resistance by improving mitochondrial biogenesis in THP-1 macrophages. Mol. Biol. Rep..

[79] Smith A.L.M., Whitehall J.C., Greaves L.C. (2022). Mitochondrial DNA mutations in ageing and cancer. Mol. Oncol..

[80] Taylor R.W., Turnbull D.M. (2005). Mitochondrial DNA mutations in human disease. Nat. Rev. Genet..

[81] Czarnecka A.M., Kukwa W., Krawczyk T., Scinska A., Kukwa A., Cappello F. (2010). Mitochondrial DNA mutations in cancer--from bench to bedside. Front. Biosci..

[82] Kopinski P.K., Singh L.N., Zhang S., Lott M.T., Wallace D.C. (2021). Mitochondrial DNA variation and cancer. Nat. Rev. Cancer.

[83] Luo Y., Ma J., Lu W. (2020). The Significance of Mitochondrial Dysfunction in Cancer. Int. J. Mol. Sci..

[84] Wallace D.C. (2012). Mitochondria and cancer. Nat. Rev. Cancer.

[85] Kim M., Mahmood M., Reznik E., Gammage P.A. (2022). Mitochondrial DNA is a major source of driver mutations in cancer. Trends Cancer.

[86] Artusi M., Nicoli S., Colombo P., Bettini R., Sacchi A., Santi P. (2004). Effect of chemical enhancers and iontophoresis on thiocolchicoside permeation across rabbit and human skin in vitro. J. Pharm. Sci..

[87] Jiang J., Li H., Qaed E., Zhang J., Song Y., Wu R., Bu X., Wang Q., Tang Z. (2018). Salinomycin, as an autophagy modulator—A new avenue to anticancer: A review. J. Exp. Clin. Cancer Res..

[88] Tan T., Li J., Luo R., Wang R., Yin L., Liu M., Zeng Y., Zeng Z., Xie T. (2021). Recent Advances in Understanding the Mechanisms of Elemene in Reversing Drug Resistance in Tumor Cells: A Review. Molecules.

[89] Wu X.Y., Xu W.W., Huan X.K., Wu G.N., Li G., Zhou Y.H., Najafi M. (2023). Mechanisms of cancer cell killing by metformin: A review on different cell death pathways. Mol. Cell. Biochem..

[90] Sirtori C.R., Castiglione S., Pavanello C. (2024). Metformin: From diabetes to cancer to prolongation of life. Pharmacol. Res..

[91] Mori S., Fujiwara-Tani R., Gyoten M., Nukaga S., Sasaki R., Ikemoto A., Ogata R., Kishi S., Fujii K., Kuniyasu H. (2023). Berberine Induces Combined Cell Death in Gastrointestinal Cell Lines. Int. J. Mol. Sci..

[92] Hojo Y., Kishi S., Mori S., Fujiwara-Tani R., Sasaki T., Fujii K., Nishiguchi Y., Nakashima C., Luo Y., Shinohara H. (2022). Sunitinib and Pterostilbene Combination Treatment Exerts Antitumor Effects in Gastric Cancer via Suppression of PDZD8. Int. J. Mol. Sci..

[93] Mori S., Kishi S., Honoki K., Fujiwara-Tani R., Moriguchi T., Sasaki T., Fujii K., Tsukamoto S., Fujii H., Kido A. (2020). Anti-Stem Cell Property of Pterostilbene in Gastrointestinal Cancer Cells. Int. J. Mol. Sci..

[94] Nishiguch Y., Fujiwara-Tani R., Nukaga S., Nishida R., Ikemoto A., Sasaki R., Mori S., Ogata R., Kishi S., Hojo Y. (2024). Pterostilbene Induces Apoptosis from Endoplasmic Reticulum Stress Synergistically with Anticancer Drugs That Deposit Iron in Mitochondria. Int. J. Mol. Sci..

[95] Kuniyasu H., Oue N., Wakikawa A., Shigeishi H., Matsutani N., Kuraoka K., Ito R., Yokozaki H., Yasui W. (2002). Expression of receptors for advanced glycation end-products (RAGE) is closely associated with the invasive and metastatic activity of gastric cancer. J. Pathol..

